# Deep convolutional neural network for reduction of contrast-enhanced region on CT images

**DOI:** 10.1093/jrr/rrz030

**Published:** 2019-05-24

**Authors:** Iori Sumida, Taiki Magome, Hideki Kitamori, Indra J Das, Hajime Yamaguchi, Hisao Kizaki, Keiko Aboshi, Kyohei Yamashita, Yuji Yamada, Yuji Seo, Fumiaki Isohashi, Kazuhiko Ogawa

**Affiliations:** 1 Department of Radiation Oncology, Osaka University Graduate School of Medicine, 2-2 Yamada-oka, Suita, Osaka, Japan; 2 Department of Radiological Sciences, Faculty of Health Sciences, Komazawa University, 1-23-1 Komazawa, Setagaya-ku, Tokyo, Japan; 3 Department of Health Sciences, Graduate School of Medicine Science, Kyusyu University, 3-1-1 Maidashi, Higashi-ku, Fukuoka, Japan; 4 Department of Oral and Maxillofacial Radiology, Osaka University Graduate School of Dentistry, 1-8 Yamada-oka Suita, Japan; 5 Department of Radiation Oncology, New York University Langone Medical Center, Laura & Isaac Perlmutter Cancer Center, 160 E 34th Street, New York, NY, USA; 6 Department of Radiation Oncology, NTT West Osaka hospital, 2-6-40 Karasugatsuji, Tennoji-ku, Osaka, Japan

**Keywords:** deep learning, convolution neural network, CT, contrast enhancement

## Abstract

This study aims to produce non-contrast computed tomography (CT) images using a deep convolutional neural network (CNN) for imaging. Twenty-nine patients were selected. CT images were acquired without and with a contrast enhancement medium. The transverse images were divided into 64 × 64 pixels. This resulted in 14 723 patches in total for both non-contrast and contrast-enhanced CT image pairs. The proposed CNN model comprises five two-dimensional (2D) convolution layers with one shortcut path. For comparison, the U-net model, which comprises five 2D convolution layers interleaved with pooling and unpooling layers, was used. Training was performed in 24 patients and, for testing of trained models, another 5 patients were used. For quantitative evaluation, 50 regions of interest (ROIs) were selected on the reference contrast-enhanced image of the test data, and the mean pixel value of the ROIs was calculated. The mean pixel values of the ROIs at the same location on the reference non-contrast image and the predicted non-contrast image were calculated and those values were compared. Regarding the quantitative analysis, the difference in mean pixel value between the reference contrast-enhanced image and the predicted non-contrast image was significant (*P* < 0.0001) for both models. Significant differences in pixels (*P* < 0.0001) were found using the U-net model; in contrast, there was no significant difference using the proposed CNN model when comparing the reference non-contrast images and the predicted non-contrast images. Using the proposed CNN model, the contrast-enhanced region was satisfactorily reduced.

## INTRODUCTION

Computed tomography (CT) imaging has been used for diagnostic imaging and planning of radiation therapy. CT examination can be conducted with images obtained both with and without a contrast medium to identify contrast-enhanced regions, to delineate the contours of structures and to calculate patient doses. Nevertheless, using contrast images changes the reading on the Hounsfield unit (HU) scale and produces streaking artifacts. This creates problems in treatment planning, leading to inaccurate dosimetry for both the target volume and the organs at risk. To overcome this problem, two sequential images are acquired without and with contrast medium. Nevertheless, the subtle differences in anatomy that are inevitable between two different time frames may change the contouring. Furthermore, taking two images is more expensive and increases the radiation dose to the patient [1, 2].

If two images are acquired in two time frames, they are acquired without contrast first and then with contrast. From the viewpoint of radiation exposure, what should be considered for the patient is the concept of ‘as low as reasonably achievable’ (ALARA) [3] as recommended by the International Commission on Radiological Protection. Nevertheless, advances in CT technology have facilitated high-speed image acquisition, and this has led to (i) more of the body being imaged at once and (ii) more CT examinations [4, 5]. An advanced CT scanner can reduce radiation exposure to patients, thereby reducing potential cancer risks [6]. Therefore, it is important to conduct CT examinations with as low a dose as possible for the required images. Consequently, the radiologist prioritizes dose reduction of the CT examination [7]. However, such low-dose CT imaging comes with an equivalently low signal-to-noise ratio [8] that degrades the images, thereby jeopardizing the possibility of detecting and diagnosing diseases. There are some techniques to address such issues using iterative reconstruction [9, 10].

Recently, dual-energy CT imaging has become available to reduce the beam hardening effect contributing to the artifacts caused by the contrast agent [11–13]. Approaches such as image acquisition with different beam energy or using energy-resolved photon-counting detectors are used to perform the dual-energy CT imaging. The former technique [14] utilizes exposure with a rapidly switched tube voltage using either a single X-ray source or a dual X-ray source; thus, the radiation exposure is higher than that of single-source CT imaging [15, 16].

Deep convolutional neural networks (CNNs) have also been used in medical imaging. Several studies have reported the practical solution of obtaining equivalent standard-dose CT images from low-dose CT images via a denoising approach using CNN [17, 18]. The purpose of these studies was not to reduce a contrast-enhanced region from a contrast-enhanced image but to map a low-dose image towards a standard-dose image by a noise reduction network. Also, Unberath *et al.* have reported an image inpainting method for digital subtraction angiography [19]. To the best of our knowledge, there have been no reports of a CNN being used to perform a contrast reduction from a contrast-enhanced CT image. When a contrast reduction using CNN would succeed, it would be possible to adapt a contrast reduction image to treatment planning for radiotherapy without non-contrast CT image acquisition for patients. The present study aims to perform a contrast reduction from a contrast-enhanced CT image.

## MATERIALS AND METHODS

### CT image acquisition

Twenty-nine patients who had been treated with radiation therapy for head and neck cancer were selected. All patient data used for analysis in the present study were obtained with written informed consent. CT images obtained with and without a contrast medium were taken sequentially. Two different scanners, a 16-detector row scanner (BrightSpeed; GE Healthcare, USA) or a 320-detector row scanner (Aquilion ONE; TOSHIBA, Japan), were used to take a CT image from the top of the head to the bottom of the trachea as the scan region. Image acquisition was performed on 15 patients using the former scanner and on the remaining 14 patients using the latter scanner. Contrast-enhanced CT image acquisition would be started 60 s after 100 ml of contrast medium is injected at an injection rate of 2 ml s^–1^ so that time between two scans (image acquisition without contrast medium followed by with contrast medium) was ~1 min. As the images were acquired for the purpose of radiation treatment, each patient was immobilized with a thermoplastic mask. The X-ray tube voltage was 120 kVp. The slice thickness of the reconstructed CT images was 2.5 mm and 2 mm in the BrightSpeed scanner and Aquilion ONE scanner, respectively. The CT-reconstructed field of view was 500 mm with 512 × 512 pixels.

### Preprocessing of CT images

**Fig. 1.**
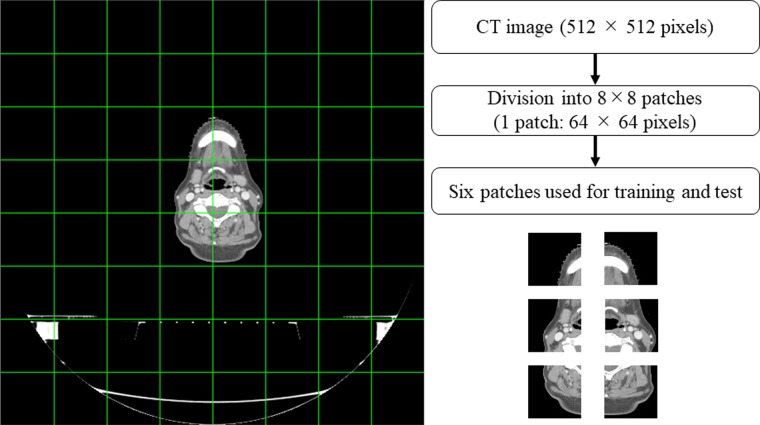
Creation process of the image patch from an original CT image.

All image data used in the present study were limited to the CT scan range from the top of the head to the bottom of the neck; images from the shoulder to the bottom of the trachea were not used. For both non-contrast and contrast-enhanced CT images, as shown in Fig. 1, the images were divided into 8 × 8 patches, i.e. each image patch had 64 × 64 pixels. Images in which a fixation device is used for patient immobilization purposes and the entire region is in air, i.e. all pixels in an image patch had a HU value of −1000, were ignored during analysis. There were 12 541 patches in total for both non-contrast and contrast-enhanced CT image pairs from the 24 patients.

### Structure of the CNN

**Fig. 2.**
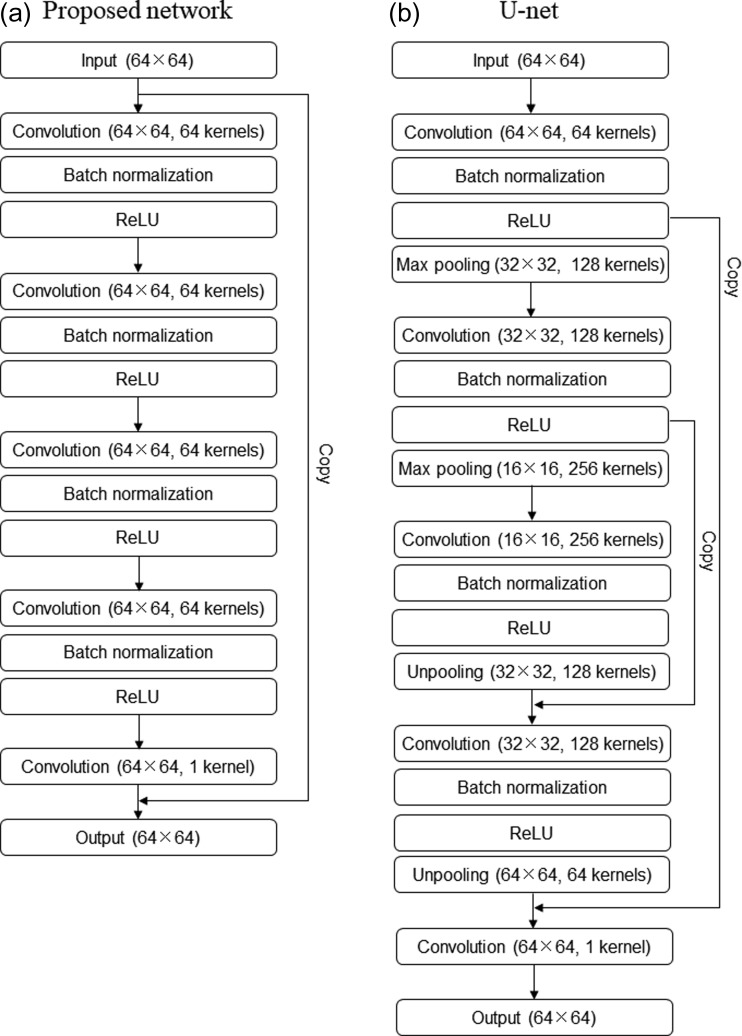
(a) Proposed convolutional neural network (CNN) structure and (b) the U-net structure. Values in parentheses denote the image size and the number of kernels.

To reduce the contrast-enhanced areas from a CT image and produce a non-contrast CT image, we use the approach of Zhang *et al.* [17] that involves using a convolutional neural network for image denoising. It was assumed that we regarded the contrast-enhanced region as the object to be processed. The proposed CNN layers are shown in Fig. 2a. The network comprises two-dimensional (2D) convolution layers, batch normalization layers, rectified linear unit (ReLU) [20] layers and, finally, a 2D convolution layer used as the image output layer for image regression. However, Chen *et al.* [16] showed that the denoising neural network approach results in overly smoothed images. Therefore, to maintain the original texture inherent in the CT image, we applied a shortcut connection to the unit with the connected path running from the image input layer to the image output layer. Such a shortcut connection is a major technique in residual learning called ResNet architecture [21], taking high-resolution features from the image input layer that can be used as extra inputs. Regarding a shortcut connection, the two trained models with and without the shortcut connection were made, and the effect of the shortcut connection on the predicted image was evaluated. Except for the final 2D convolution layer, the other 2D convolution layers had 3 × 3 pixels of kernel size, 64 filters, 1 × 1 pixels of stride size and 1 × 1 pixels of zero-padding such that the output image size was the same as the input image size in each 2D convolution layer. The batch normalization layers [22] were used to promote a training process that generally expects convergence. The ReLU layers provided the activation function.

For comparison, we referred to the U-net architecture [20] proposed in Ronneberger *et al.* and made the network architecture shown in Fig. 2b. The U-net has two main flows as encoding and decoding parts. We set several layers as encoding parts, which comprise the 2D convolution layer, the batch normalization layer, the ReLU layer and the max pooling layer. These layer units were repeated twice as encoding parts. As decoding parts, the unit including the 2D convolution layer, the batch normalization layer, the ReLU layer and the unpooling layer was repeated twice. Regarding the feature of the U-net architecture, the two shortcut connections from the ReLU layer in the encoding part to the decoding part were set so that the high-resolution features from the first part can be succeeded to the extra input for the convolution layer in the encoding parts. Finally, a 2D convolution layer was used as the image output layer for image regression. A 2D convolution layer had 3 × 3 pixels of kernel size, 1 × 1 pixels of stride size and 1 × 1 pixels of zero-padding. As for the max pooling layer, 2 × 2 pixels of pool size and 2 × 2 pixels of stride size were set so that the image input size was down-sampled in half. In contrast, as for the unpooling layer, 4 × 4 pixels of pool size, 2 × 2 pixels of stride size and 1 × 1 pixels of cropping size were set so that the image input size was up-sampled in duplicate. The number of kernels in a 2D convolution layer started at 64, and then increased 2-fold according to the down-sampling process, and decreased by half according to the up-sampling process.

### Training

The pixel data used in the training process were the original pixel values in HU from the CT image. The kernel parameters of the 2D convolution layer were estimated for training by minimizing the following objective function. We considered the training data set $T=\{(x_{1},y_{1}),(x_{2},y_{2}),\ldots \;\;\ldots \;\;\ldots ,\;(x_{n},y_{n})\}$, where $\left\{x_{k}\right\}$ and $\left\{y_{k}\right\}$, *k* = 1, 2,…, *n* denote non-contrast and contrast-enhanced image patches, respectively, and *n* is the total number of training samples. The mini-batch number was set to 100, and the mini-batch patches were randomly selected in the training data for each iteration. The object function was the loss function *L* that we defined as a root mean square error, namely

(1) $$L\left(T\right)=\sqrt{\frac{1}{100}\overset{100}{\underset{i=1}{\sum }}∥x_{i}-P\left(y_{i}\right){∥}^{2}},$$

where $P\left(y_{i}\right)$ denotes the predicted image patch of image *i*. The loss function was optimized using the adaptive moment estimation proposed by Kingma *et al.* [23] Training was performed using MATLAB R2018a on a laptop computer (NVIDIA GeForce GTX 1060 GPU, 6 GB RAM). The learning rate was initially set to 0.001, and was changed by one-tenth at 10 epochs each. Maximum epochs were set to 100.

### Cross-validation of the CNN

The performance of both the proposed CNN model and the U-net model was evaluated using a 6-fold cross-validation procedure. First, 29 patients were separated into 24 patients and 5 patients. All the image patches in 24 patients were used for the training and validation process. A total of 12 541 patches were divided randomly into six equal-sized groups. At each training, five groups were used as training data (10 450 patches) to train two models, and the other group was used as validation data (2091 patches). Regarding the test process of two models, five patients who were not used for training and validation processes were used. The number of image patches was 2182.

### Quantitative evaluation

**Fig. 3.**
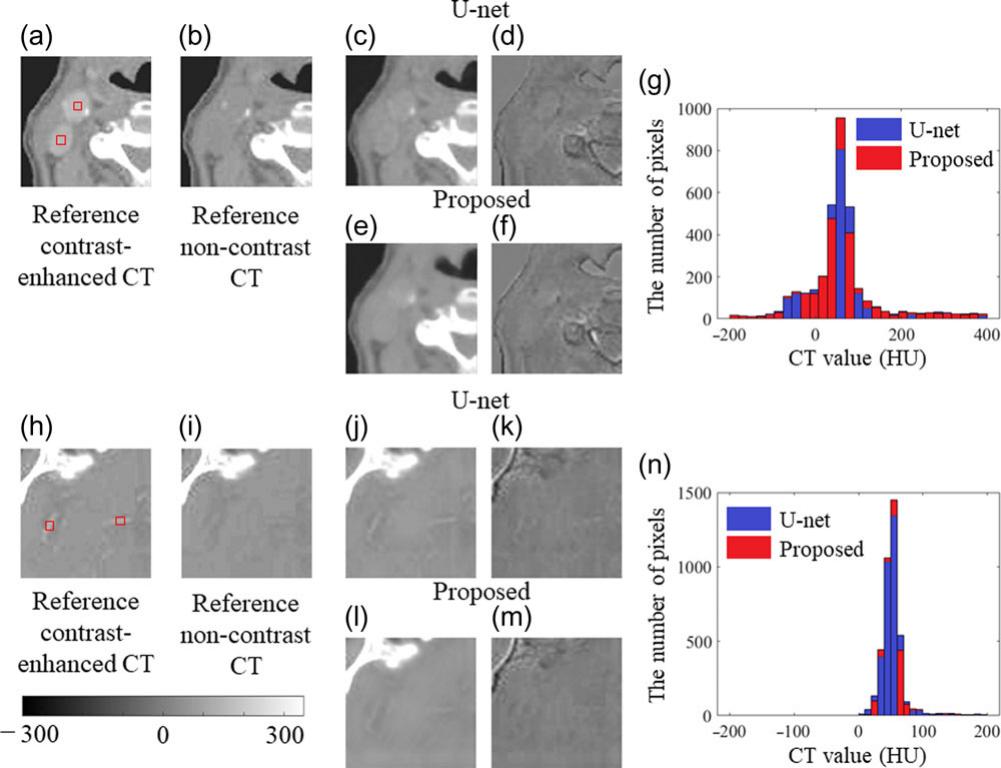
Qualitative comparison of predicted non-contrast images against reference non-contrast images. First column: (a) and (h) reference contrast-enhanced images; second column: (b) and (i) reference non-contrast images. Red squares denote contrast-enhanced regions. Third column: (c), (e), (j) and (l) images predicted with the U-net model and the proposed CNN model. Fourth column: (d), (f), (k) and (m) images subtracted between each predicted image and reference non-contrast image. The grayscale bar range is from −350 to 350. Fifth column: (g) HU histogram of the non-contrast images (c) predicted by the U-net model and HU histogram of the non-contrast image (e) predicted by the proposed CNN model. (n) HU histograms of the non-contrast image (j) predicted by the U-net model and those of the non-contrast image (l) predicted by the proposed CNN model. The bin width on the horizontal axis is 10 HU. The vertical axis gives the number of pixels.

As shown in red squares in Fig. 3a and h, 50 regions of interest (ROIs) were manually selected on the reference contrast-enhanced image of the test data under a window width of 500 and a window level of 0. These ROIs were placed on blood vessels with diameters ranging between 3 and 15 mm, and the mean pixel value of the ROIs was calculated. The size of the ROIs was set to 4 × 4 pixels. The mean pixel values of the ROIs at the same location on the reference non-contrast image and the non-contrast image predicted by the proposed CNN model and the U-net model were calculated. To evaluate the capability for reduction of contrast enhancement, the mean pixel value of 50 ROIs in the reference contrast-enhanced images was compared with that in the predicted non-contrast image using the proposed CNN model and U-net model. To know how the predicted non-contrast image was close to the reference non-contrast image, the mean pixel value of 50 ROIs in the predicted non-contrast images was compared with that of the reference non-contrast images. The final mean pixel value was averaged by the mean pixel value of the six models cross-validated in each proposed CNN model and the U-net model.

In addition to the aforementioned comparison, differences in the overall image quality of the non-contrast CT images predicted by the proposed model and U-net model were evaluated. The HU histogram for all voxels in non-contrast CT images predicted using test data of 2182 patches was calculated. The bin width of the CT values was set to 10 HU. The range of evaluated CT values was −200 to 400 HU.

Regarding statistical analysis, the normality of the data was checked with the Shapiro–Wilk test, and comparison analyses used the two-tailed paired *t*-test or the Wilcoxon signed-rank test as appropriate. A *P*-value <0.05 was considered statistically significant. The statistical analysis was performed using version 3.1.2 of R statistical software (R Foundation, Austria).

### Dosimetric evaluation

**Fig. 4.**
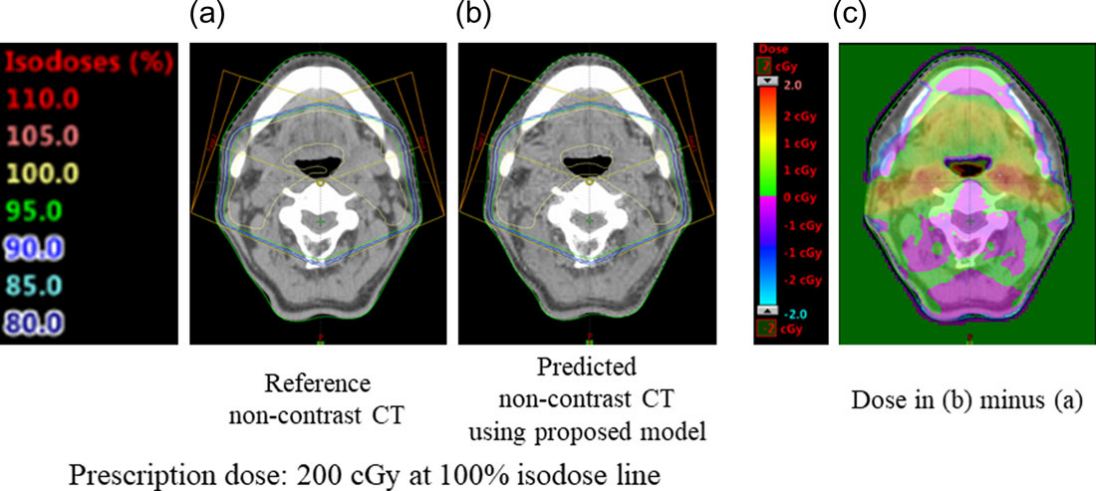
Comparison of (a) dose distributions calculated in the reference non-contrast CT and (b) dose distributions calculated in the predicted non-contrast CT using the proposed CNN model. (c) Dose difference between (b) and (a). The prescribed dose is 200 cGy. The dose in color bars ranges between −2 cGy and 2 cGy.

One out of five test patients was selected for dosimetry evaluation. Contrast reduction was performed using the proposed CNN model on the image patches from this patient. The predicted non-contrast image patches were rearranged to obtain the size of the original CT image, i.e. 512 × 512 pixels. A treatment plan was generated anticipating the use of two non-opposed beams of 6 MV X-ray with an enhanced dynamic wedge of 15°. The Eclipse version 13.4 (Varian Medical Systems, Palo Alto, CA, USA) treatment planning system was used, and the anisotropic analytical algorithm (version 13.7.14) was used to correct for dose inhomogeneities. The grid resolution for the dose was 2 mm. First, a treatment plan was created with the reference non-contrast CT images and dose calculations were performed as shown in Fig. 4a. The prescribed dose was 200 cGy at the isocenter. Furthermore, the same treatment plan (i.e. the same monitor units) was superimposed on the predicted non-contrast CT images, and dose calculations were performed as shown in Fig. 4b. The dose difference between the predicted non-contrast CT images and the reference non-contrast CT images was then calculated, and is shown in Fig. 4c.

## RESULTS

### Computation time

**Fig. 5.**
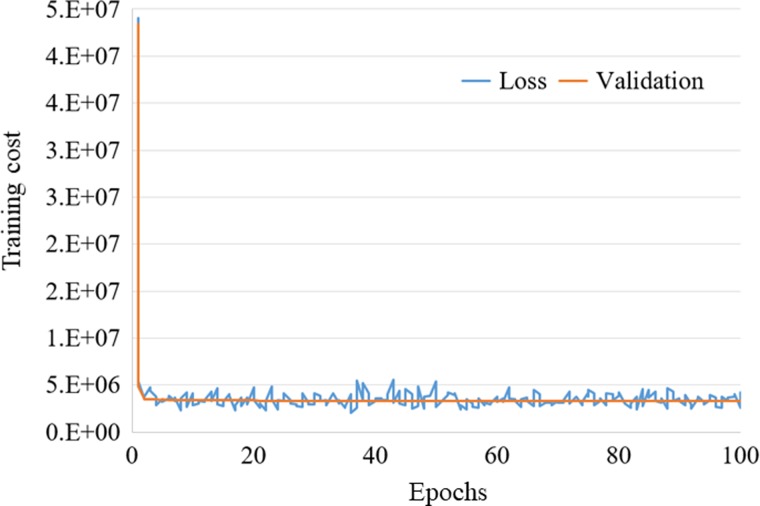
Training cost curve as a function of epochs. The blue line presents the training loss curve. The orange line presents the validation loss curve.

Because the number of training data was 10 450 patches, one epoch required 105 iterations to cover all training samples using the mini-batch number of 100. We found empirically that 100 epochs were required for both models to converge. Figure 5 shows a sample training cost curve as a function of epochs. The blue line presents the training loss curve and the orange line presents the validation loss curve. The computation time took ~37 min and 35 min for the proposed CNN model and the U-net model, respectively, in our computing environment. Once trained, the model took ~10 s to generate the non-contrast image patches from the contrast-enhanced image patches for all 2182 test image patches.

### Qualitative and quantitative evaluations of predicted non-contrast images

**Fig. 6.**
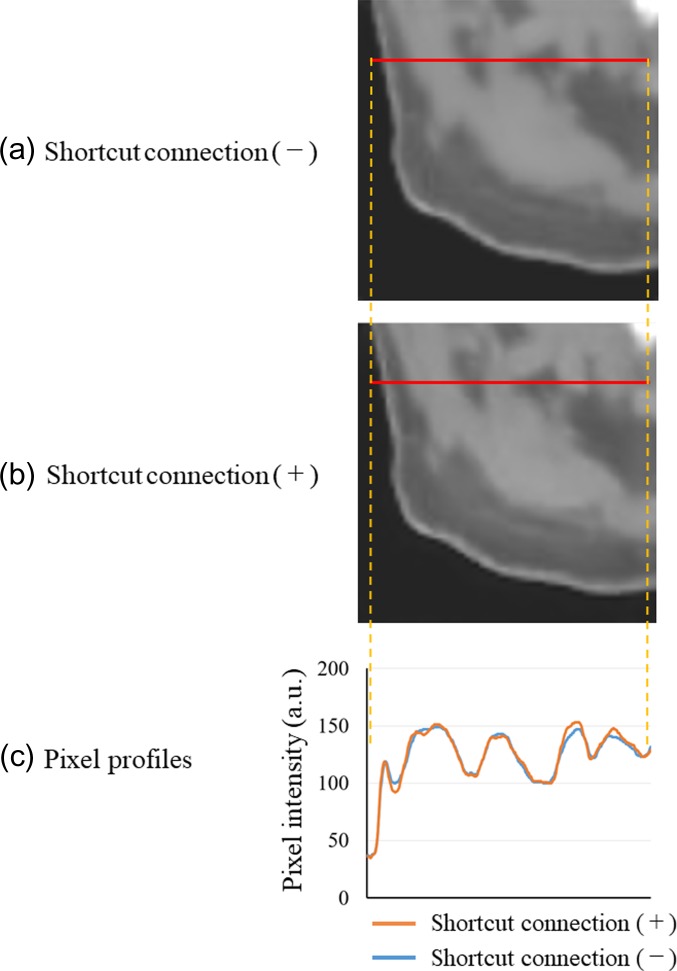
Effects of the shortcut connection in the proposed CNN model. (a) Predicted non-contrast image with no shortcut connection. (b) Predicted non-contrast image with a shortcut connection. (c) Comparison of pixel intensity profiles at the red line marked in images (a) and (b).

The predicted image results for a sample patch with and without a shortcut connection in the proposed CNN model is shown in Fig. 6. The non-contrast image patch predicted with no shortcut connection is shown in Fig. 6a, and the non-contrast image path predicted with a shortcut connection is shown in Fig. 6b. Comparisons of the pixel intensity profiles at the red line in the two images are shown in Fig. 6c. The pixel intensity is relatively enhanced at the border between low- and high-density tissue, such as the border between fat and muscle, when the shortcut connection is used.

The predicted image results for two sample image patches, which include large blood vessels on the upper row and small blood vessels on the lower row, are shown in Fig. 3 in terms of the U-net model and the proposed CNN model. The two images in the first column are the contrast-enhanced (Fig. 3a and h) images used for reference. In the second column, the reference non-contrast images are shown in Fig. 3b and i. In the third column, the non-contrast images predicted by the U-net model and the proposed CNN model are shown in Fig. 3c, e, j and l, respectively. In the fourth column, the subtraction images of the predicted images against the reference non-contrast images are shown in Fig. 3d, f, k and m, respectively. For all images, the window level was set to zero and the window width was set to between −350 and 350 for visualization. In the predicted images with large blood vessels, the contrast-enhanced regions presented in red squares in Fig. 3c and e were reduced. In contrast, in the predicted images with small blood vessels, the contrast-enhanced regions were still shown in Fig. 3j using the U-net model. Regarding Fig. 3l using the proposed CNN model, the predicted non-contrast image (Fig. 3l) was visually comparable to the reference non-contrast image (Fig. 3i). Although the visual intensity in the bone was not changed as shown in Fig. 3d, f, k and m, the subtle change in intensity at the interface between soft tissue and bone was found. Figure 3g and n shows the HU histograms of the non-contrast images predicted by the proposed CNN model and the U-net model, respectively. A bin width of CT value was set to 10 HU. Figure 3g shows the large blood vessels in an evaluated CT value range between −200 HU and 400 HU, and Fig. 3n shows the small blood vessels in an evaluated CT value range between −200 HU and 200 HU. The HU range focuses on emphatically making the difference in visibility of soft tissue. Recognizing the difference between two models from these two figures was difficult.

**Fig. 7.**
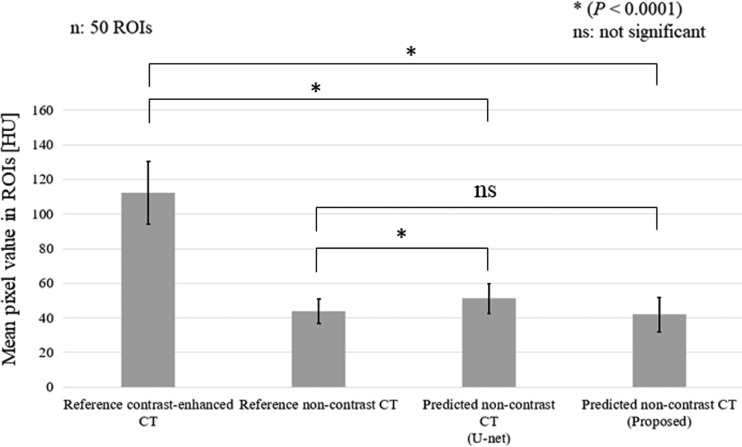
Comparisons of mean pixel values at the 50 ROIs in the reference contrast-enhanced image, reference non-contrast image, predicted non-contrast image (U-net model) and predicted non-contrast image (proposed CNN model).

In terms of a quantitative comparison, the mean pixel values of the 50 ROIs selected manually on the reference contrast-enhanced image, those of the reference non-contrast image and those of the predicted non-contrast image for both the U-net model and the proposed CNN model were calculated as shown in Fig. 7; error bar denotes 1 SD. In terms of the contrast-enhanced reduction, the mean pixel value of the reference contrast-enhanced image was 112. Both the U-net model and the proposed CNN model significantly reduced the contrast-enhanced regions (*P* < 0.0001). The mean pixel values in the U-net model and the proposed CNN model were 51 and 42, respectively. Nevertheless, regarding the comparison between the reference non-contrast image and the predicted non-contrast image, significant differences (*P* < 0.0001) in pixels were found for the U-net model. The mean pixel value in the U-net model, i.e. 51, was greater than that in the reference non-contrast image, i.e. 44. In contrast, no significant difference was found for the proposed CNN model.

**Fig. 8.**
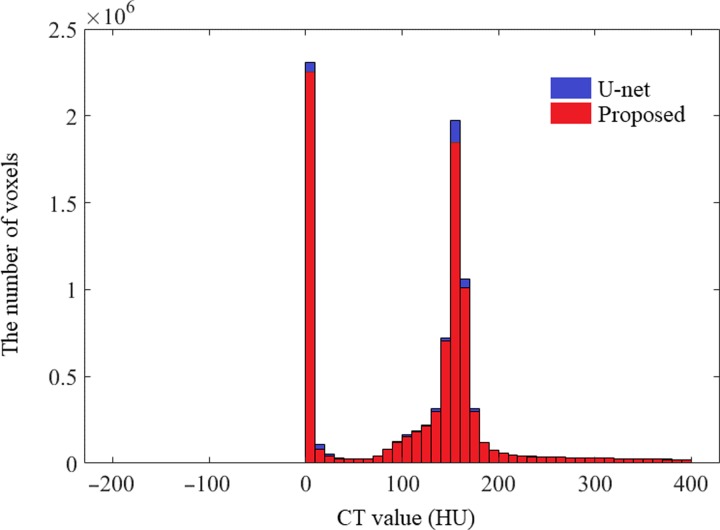
HU histogram of non-contrast images of all test data with 2182 patches predicted by the U-net model and that of the non-contrast image predicted by the proposed CNN model. The bin width on the horizontal axis is 10 HU. The vertical axis gives the number of pixels.

To better understand the differences in overall image quality of the non-contrast CT images predicted by the proposed model and the U-net model, the HU histogram for all voxels in the predicted non-contrast image using test data of 2182 patches is shown in Fig. 8. As the significant difference appears in the U-net model shown in Fig. 7, more voxels are included in the U-net model than those in the proposed CNN model.

### Dosimetric evaluation

Figure 4a and b shows dose distributions predicted from two non-opposed beams with an enhanced dynamic wedge of 15°. These distributions were calculated on the reference non-contrast CT image and the predicted non-contrast CT image using the proposed CNN model. Although the differences are difficult to discern visually using the isodose lines, a subtle dose difference in cGy can be seen in Fig. 4c. The color bar ranges between −2 cGy and 2 cGy, and the prescription dose was 200 cGy; thus, the dose difference in the irradiated region is as small as 1%.

## DISCUSSION

We proposed here a CNN structure for reducing the contrast-enhanced region from contrast-enhanced CT images, and compared it with another CNN model named the U-net. The image pairs for training and testing were made from image patches based on the same method as shown in the studies conducted by Nishio *et al.* [15] and Chen *et al.* [16] Regarding results with and without the shortcut connection, as shown in Fig. 6, the interface between muscle and fat regions is blurred without the shortcut connection, and this effect is also found in Fig. 6c in the comparison of pixel profiles. This observation agrees with the report of Chen *et al.* [16] Because the non-contrast CT image predicted by the proposed CNN model is desired to be as close as possible to the reference CT image in terms of overall image quality, the shortcut connection proves helpful.

Given that large image data sets are generally necessary for training, we showed that it is possible to create a large data set efficiently by chopping the original images into patches. Notably, the image patches chopped from two neighboring slices can be close to each other. Therefore, the training image patches were selected randomly and were gathered to form a mini-batch over the iterative process of training. Nishio *et al.* used a CT image divided into patches comprising 28 × 28 pixels to increase the number of training images and to validate their patch-based image denoising method [15]. Chen *et al.* [16] found that image patches are useful for training instead of whole images for two main reasons. First, local structures can be well represented by image patches. Secondly, deep learning requires a large training data set, and chopping the original images into patches can efficiently increase the number of samples. The original image was chopped into patches for our training process. In case the training uses the original images alone, i.e. 512 × 512 pixels, as the relative location of the body with respect to the image in an axial slice is considered in the training process, preprocessing with image chopping can effectively mask the locations of tissue such as fat, muscle and bone within an image patch. To address this concern, all image data from the top of the head to the bottom of the trachea could be used when image-chopping preprocessing is applied to training data. The image-chopping technique was applied herein to boost the number of samples, and the proposed CNN model effectively reduces the contrast-enhanced regions in the images. However, the training of the neural network could be improved more effectively by increasing the number of patients who provided training data. The number of patients is certainly a limitation herein, and we plan to increase the number of patients in the data set in the future.

Regarding visual inspection of the potential reduction in the contrast-enhanced region shown in Fig. 3, it was difficult to detect any difference except in Fig. 3j between the U-net model and the proposed CNN model. Nevertheless, regarding the quantitative analysis shown in Fig. 7, we found that the proposed CNN model was suitable because there was no significant difference in mean pixel value between the predicted and reference image under non-contrast imaging. In contrast, we determined that the mean pixel values in the ROIs of the contrast-reduced CT images predicted using the U-net model were significantly greater than those of the reference non-contrast CT images. As shown in Fig. 8, more voxels were included in the U-net model than those in the proposed CNN model with the range of CT values (0–180) that are regarded as soft tissue. When calculating the mean pixel values of the ROIs, the size of the ROIs was set to 4 × 4 pixels, which corresponds to ~4 mm × 4 mm. Because the diameter of blood vessels ranges between 3 mm and 15 mm, we chose the size of the ROI to cover the whole range from small blood vessels to large blood vessels.

From the dosimetric evaluation shown in Fig. 4c, the dose distribution calculated with the non-contrast CT image predicted by the proposed CNN model was within 1% of the distribution calculated with the reference non-contrast CT image. Two non-opposed beams were used to enhance the impact of dosimetry because when four fields or more are used in a treatment plan, such as intensity-modulated radiation therapy, the beam weight will be divergent, which reduces the impact of dosimetry through a beam across the predicted non-contrast region.

Deeper CNN models are difficult to train. Herein, we empirically set a depth of the CNN model as shown in Fig. 1. Although errors in conventional networks tend to increase with the high increase in network depth, there is a report that sophisticated networks such as ResNet [21] could achieve high accuracy with deeper layers than conventional networks. In future work, other CNN architectures should be tested and compared with the proposed CNN model for reducing the contrast-enhanced regions in CT images.

Regarding computation time, reducing the contrast-enhanced region from the contrast-enhanced image required 10 s for 2182 image patches. Because the average number of image patches per patient was ~440, we estimate that it would require 2 s to perform contrast-enhanced reduction per patient, i.e. the imaging range between the top of the head and the bottom of the neck. Once the CNN is trained, the prediction process occurs quickly (in a few seconds), as reported elsewhere [24].

In conclusion, a CNN structure using a residual network was proposed and compared with the U-net model for the evaluation to reduce contrast-enhanced region. The proposed CNN model was shown to be suitable for reducing the contrast-enhanced regions in CT images. The proposed CNN network helps reduce radiation exposure for patients and is able to predict non-contrast CT images, which are available to use in a treatment plan for dose calculation with an accuracy of 1%. In future work, we will apply the present CNN structure to other portions of the body.
